# The effect of hepatitis B virus on the risk of pregnancy outcomes: a systematic review and meta-analysis of cohort studies

**DOI:** 10.1186/s12985-023-02182-0

**Published:** 2023-09-14

**Authors:** Maryam Afraie, Ghobad Moradi, Kamran Zamani, Mobin Azami, Yousef Moradi

**Affiliations:** 1 Student Research Committee, Kurdistan University of Medical Sciences, Sanandaj, Iran; 2 Department of Epidemiology and Biostatistics, Faculty of Medicine, Kurdistan University of Medical Sciences, Sanandaj, Iran; 3 Social Determinants of Health Research Center, Research Institute for Health Development, Kurdistan University of Medical Sciences, Sanandaj, Iran

**Keywords:** Hepatitis B virus, Pregnancy outcomes, Pre-eclampsia, Gestational diabetes, Abortion, Premature birth, Infant death

## Abstract

**Background:**

The effect of HBV on neonatal and maternal outcomes can create a basis for more accurate clinical decision-making. So, the aim of this meta-analysis is to detrmine the effect of chronic hepatitis B virus on the risk of pregnancy outcomes by combining cohort studies.

**Methods:**

International databases in this meta-analysis included the Cumulated Index to Nursing and Allied Health Literature (CINAHL), SPORT Discuss via the EBSCO interface, PubMed (Medline), Scopus, Web of Science, Embase, which were searched up to April 2023. All cohort studies reporting the risk ratio (RR) with a 95% confidence interval (CI) were included in the study. The quality assessment was done based on the Newcastle–Ottawa Scale (NOS).

**Results:**

Finally, thirty-five cohort studies were selected for meta-analysis. Outcomes of interest included pre-eclampsia, gestational diabetes, abortion, preterm birth, infant death, and other related outcomes. Results showed that the pooled RR for incident gestational diabetes in pregnant women with choronic hepatitis B infection was 1.16 (RR: 1.16; 95% CI 1.13–1.18; I-square: 92.89%; *P* value: 0.00). Similarly, the association between the presence of hepatitis B infection in pregnant women and the occurrence of pre-eclampsia was 1.10 (RR: 1.10; 95% CI 1.04–1.16; I-square: 92.06%; *P* value: 0.00). The risk of preterm delivery in pregnant women with hepatitis B infection was 1.17 times that of pregnant women without hepatitis B infection (RR: 1.17; 95% CI 1.14–1.20; I-squared: 94.32%; *P* value: 0.00).

**Conclusion:**

This meta-analysis found that hepatitis B infection during pregnancy may be associated with an increased risk of gestational diabetes, preterm delivery, pre-eclampsia, and eclampsia. However, confirmation of this association, as well as the specific biological pathways involved in the association between HBV infection and pregnancy outcomes, requires further investigation.

**Supplementary Information:**

The online version contains supplementary material available at 10.1186/s12985-023-02182-0.

## Introduction

A double-stranded DNA virus belonging to the hepadnaviridae family, hepatitis B virus (HBV). The virus is enveloped and has a core with a viral DNA genome of approximately 3200 base pairs. In patient blood, the virus was initially identified as “Australia antigen,” subsequently known as hepatitis B surface antigen (HBsAg). Later on, as a marker for those at high risk of transmitting the disease, the hepatitis B e antigen (HBeAg) was discovered. The virus penetrates a hepatocyte, delivers its genome to the nucleus, and changes the relaxed circular DNA into covalently closed circular DNA (cccDNA) [1–3]. Significant human morbidity and death are brought on by HBV infection, mostly as a result of the effects of chronic infection. According to recent estimates of chronically infected people ranging from 240 to 350 million, more than two billion people have ever been infected with HBV [4–6]. Around 0.5–1.2 million people die annually on average [7]. There are three geographic regions where the prevalence of HBV infection is highest: East Asia and Africa (> 8%), the Mediterranean region (2–8%), and Eastern Europe (2%) [8]. More than half of the 350 million HBV carriers worldwide acquire the virus during pregnancy; rates of mother-to-child transmission also differ dramatically depending on the mother’s hepatitis B e antigen (HBeAg) status [9, 10]. Some mechanisms explain how HBV infection affects pregnancy. Reduced CD8 T cells and increased viral activity are caused by inhibiting the Th1 immune response and simulating the Th2 immune response, impairing the immunological response to HBV [11]. Due to the cross-reaction, increased regulatory T cells, and malfunctioning CD8 T cells, the exposure of the fetus to HBeAg may generate fetal T helper cell tolerance to HBeAg and HBcAg, which rasie HBV DNA levels during pregnancy [12, 13]. Studies have shown an increased risk of both newborn and maternal morbidity associated with HBV infection, including fetal distress, gestational diabetes mellitus, preterm delivery, and meconium peritonitis[14–18]. Also, antepartum hemorrhage causing placental abruption and placenta previa can increase. A lower Apgar score is the only perinatal complication [14, 18]. However, there isn’t much research on the mechanisms underlying these results [19].

The effect of HBV on neonatal and maternal outcomes can create a basis for more accurate clinical decision-making. By identifying the specific connection between HBV and pregnancy outcomes, clinicians and specialists can reduce the impacts and enhance the quality of life for HBV patients. The research may aid in updating clinical guidelines and improving the care of HBV patients, which may aid in the early detection and prevention of pregnancy outcomes. Thus, by combining cohort data, we aimed to systematically review and meta-analyze the relationship between HBV and pregnancy outcomes.

## Methods

The current study was a systematic review and meta-analysis, which was conducted to determine the exact relationship between the presence of infection and the chronic HBV and the occurrence of maternal outcomes such as pre-eclampsia, gestational diabetes, abortion, premature birth, infant death, and other related results. The primary studies in this meta-analysis were prospective or retrospective cohort studies. All the steps of this study were developed and carried out based on the structure of the Preferred Reporting Items for Systematic Reviews and Meta-Analyses (PRISMA) guideline [20].

At first, the search strategy aimed at retrieving cohort studies published in the target field in international databases such as the Cumulated Index to Nursing and Allied Health Literature (CINAHL), SPORT Discuss via the EBSCO interface, PubMed (Medline), Scopus, Web of Science, and Embase. Then the retrieved articles were screened in Endnote version 8. Keywords were included: “Hepatitis B”, “Hepatitis B Virus”, “Pregnancy Outcome”, “Maternal Morbidity”, “Maternal Death”, “Pre-Eclampsia”, “Premature Birth”, and “Gestational Diabetes”, along with their synonyms in the Mesh database. To perform the screening, first, the duplicates of retrieved articles in the software were removed, and then, in the first step, screening based on the title; in the second step, screening based on the subject; and in the final step, screening based on the full text of the articles was done. And after these steps, the final articles were selected. The time frame for searching international databases and screening was from January 1990 to February 2023. Also, all the screening steps were done independently by authors (MA and MA), and disputes were resolved by the third researcher (YM), who was an expert. To carry out a comprehensive and detailed search, or in other words, to carry out gray literature to complete the search strategy, the first ten pages of Google Scholar along with a manual search (checking the sources and references of the final selected studies) were also done by the authors. The inclusion criteria and the final selection of studies in this meta-analysis were based on the PECOT structure, the specifics of which are detailed in Table 1. The studies that had all the characteristics listed in Table 2 were included in the present meta-analysis. After the screening, to extract the desired information from these articles based on the purpose of the study, a checklist prepared with the opinion of experts was used. Checklist elements included authors’ names, type of study, year of publication, total sample size, country of study, type of population, age, and effect size reported in the studies. Two authors (MA and MA/KZ) carried out data extraction independently, and the third researcher (YM), an expert, resolved disputes.

**Table 1:** Selection criteria for primary studies to enter the present meta-analysis

P (Population)	E (Exposure)	C (Comparison)	O (Outcomes)	T (Type of Studies)
The target population included all pregnant women without any restrictions	The intended exposure or cause was the presence of infection or hepatitis B virus in pregnant women	The comparison group included healthy pregnant women without hepatitis B infection	The intended Outcomes included neonatal death, miscarriage, eclampsia or preeclampsia, preterm birth, gestational diabetes, and other outcomes reported in selected cohort studies	The type of studies included only cohort studies

**Table 2:** The charactristics of included studies related to effect of HBV on the risk of maternal outcomes

References	Authors (Years) Country	Type of study	Study population	Sample size	Neonatal death	Eclampsia	Number of Pre-Eclampsia (Positive pregnancies)	Maternal age (Yrs, mean ± SD)	Gestational age (weeks, mean ± SD)	Gestational hypertension	Parity	GDM	Preterm birth
[38]	Zhang (2020) China	Retrospective cohort	HBsAg-positive and HBsAg-negative pregnancies	HBsAg-positive = 9699 HBsAg-negative = 73,076	NR	NR	HBsAg-positive = 139 (1.43%) HBsAg-negative = 1302 (1.78%)	HBsAg-positive = 30.33 ± 4.50 HBsAg-negative = 30.28 ± 4.45	HBsAg-positive = 38.18 ± 2.96 HBsAg-negative = 38.17 ± 3.51	HBsAg-positive = 157 HBsAg-negative = 1421	1 = HBsAg-positive = 82 (0.85%) HBsAg-negative = 760 (1.04%) > 1 = HBsAg-positive = 9617 (99.15%) HBsAg-negative = 72,316 (98.96%)	HBsAg-positive = 1663 HBsAg-negative = 11,982	NR
[22]	Tan (2016) China	Retrospective cohort	HBsAg-positive and HBsAg-negative pregnancies	HBsAg-positive = 948 HBsAg-negative = 21,426	NR	NR	HBsAg-positive = 33 (3.5%) HBsAg-negative = 523 (2.4%)	< 35 = HBsAg-positive = 813 (85.8) HBsAg-negative = 18 876 (88.1) HBsAg-positive = 135 (14.2) HBsAg-negative = 2550 (11.9)	39 (38–39)	HBsAg-positive = 9 HBsAg-negative = 288	Nulliparity = HBsAg-positive = 713 (75.2) HBsAg-negative = 16 489 (77.0) Multiparity = HBsAg-positive = 235 (24.8) HBsAg-negative = 4937 (23.0)	HBsAg-positive = 112 HBsAg-negative = 1758	NR
[21]	Stokkeland (2017) Sweden	Retrospective cohort	HBsAg-positive and HBsAg-negative pregnancies	HBsAg-positive = 2990 HBsAg-negative = 1,090,979	NR	NR	HBsAg-positive = 59 (1.97) HBsAg-negative = 30,030 (2.75)	≤ 24 = HBsAg-positive = 506 HBsAg-negative = 156,906 25–34 = HBsAg-positive = 1780 HBsAg-negative = 709,970 ≥ 35 = HBsAg-positive = 7.4 HBsAg-negative = 226,587	NR	NR	1 = HBsAg-positive = 1014 HBsAg-negative = 488,887 2 = HBsAg-positive = 989 HBsAg-negative = 396,034 + 3 = HBsAg-positive = 987 HBsAg-negative = 206,058	HBsAg-positive = 68 HBsAg-negative = 11,262	HBsAg-positive = 175 HBsAg-negative = 53,452
[29]	Lok (2021) Hong Kong	Retrospective cohort		HBV negative = 79487HBV positive = 8402	HBV negative = 105 HBV positive = 11	HBV negative = 37 HBV positive = 5	HBsAg-positive = 66 (0.78%) HBsAg-negative = 1103 (1.25%)	NR	NR	HBsAg-positive = 185 HBsAg-negative = 2366	Nulliparity = HBsAg-positive = 4082 HBsAg-negative = 39,178 Multiparity = HBsAg-positive = 4320 HBsAg-negative = 40,309	HBsAg-positive = 763 HBsAg-negative = 6877	HBsAg-positive = 522 HBsAg-negative = 5299
[25]	Chen (2022) United States	Retrospective cohort study	HBsAg-positive and HBsAg-negative pregnancies	Control = 28,499,085 HBV = 51,200	NR	Control = 5.28% HBV = 4.48%		NR	NR	NR	NR	Control = 6.94% HBV = 12.94%	Control = 6.27% HBV = 5.52%
[37]	Yin (2021) China	Retrospective cohort study	HBsAg-positive and HBsAg-negative pregnancies	HBV negative = 36,500 HBV positive = 3039	NR	NR	HBV negative = 924 HBV positive = 103	NR	38.55 ± 2.17	HBV negative = 242 HBV positive = 18	NR	HBV negative = 3529 HBV positive = 366	HBV negative = 1720 HBV positive = 119
[23]	Bajema (2018) United States	Retrospective cohort study	HBsAg-positive and HBsAg-negative pregnancies	HBV negative = 22 410 HBV positive = 4391	NR	HBV negative = 25 HBV positive = 9	HBV negative = 1299 HBV positive = 177	NR	NR	NR	0 = HBsAg-positive = 1935 HBsAg-negative = 9249 > 1 = HBsAg-positive = 2388 HBsAg-negative = 12,790	HBV negative = 1109 HBV positive = 389	NR
[36]	Xiong (2021) China	Retrospective cohort study	HBsAg-positive and HBsAg-negative pregnancies	HBV negative = 6,216 HBV positive = 795	NR	NR	HBV negative = 132 HBV positive = 16	HBV negative = 31 (28–34) HBV positive = 31 (28–34)	NR	NR	NR	HBV negative = 1585 HBV positive = 221	HBV negative = 967 HBV positive = 141
[27]	Connell (2011) USA	Retrospective cohort study	HBsAg-positive and HBsAg-negative pregnancies	HBV negative = 1 668 911 HBV positive = 1458	NR	NR	HBV negative = 4.46% HBV positive = 4.25%	NR	NR	HBsAg-negative = 57,744 HBsAg-positive = 40	NR	HBsAg-negative = 11,849 HBsAg-positive = 29	HBsAg-negative = 147,532 HBsAg-positive = 132
[18]	Lao (2007) China	Retrospective cohort study	HBsAg-positive and HBsAg-negative pregnancies	HBV negative = 12,547 HBV positive = 1138	NR	NR	HBV negative = 2.8% HBV positive = 1.8%	HBsAg-negative = 30.3 ± 5.3 HBsAg-positive = 30.2 ± 5.2	HBsAg-negative = 39.0 ± 1.8 HBsAg-positive = 38.9 ± 1.8	NR	NR	HBsAg-negative = 1279 HBsAg-positive = 141	
[28]	Lao (2013) Hong Kong	Retrospective cohort study	HBsAg-positive and HBsAg-negative pregnancies	HBV negative = 77,936 HBV positive = 8636	NR	NR	HBV negative = 1.1% HBV positive = 0.8%	HBsAg-negative = 29.9 ± 5.2 HBsAg-positive = 29.8 ± 5.1	HBsAg-negative = 39.0 ± 1.8 HBsAg-positive = 38.9 ± 1.8	HBsAg-negative = 545 HBsAg-positive = 52	NR	HBsAg-negative = 5221 HBsAg-positive = 553	HBsAg-negative = 4988 HBsAg-positive = 526
[50]	Lobstein (2011) Germany	Retrospectively	HBsAg-positive and HBsAg-negative pregnancies	HBV negative = 8,154 HBV positive = 39	NR	HBV negative = 26 HBV positive = 0	HBV negative = 160 HBV positive = 1	NR	NR	NR	NR	HBsAg-negative = 36 HBsAg-positive = 0	HBsAg-negative = 922 HBsAg-positive = 8
[31]	Reddick (2011) United States	Retrospective cohort study	HBsAg-positive and HBsAg-negative pregnancies	HBV negative = 296,773 HBV positive = 891	NR	NR	HBV negative = 9729 HBV positive = 33	NR	NR	NR	NR	HBV negative = 7464 HBV positive = 35	HBsAg-negative = 35,991 HBsAg-positive = 197
[32]	Sirilert (2014) Thailand	Retrospective cohort study	HBsAg-positive and HBsAg-negative pregnancies	HBsAg-negative = 22,331 HBsAg-positive = 1472	NR	NR	HBsAg-negative = 1496 HBsAg-positive = 91	HBV negative = 27.82 ± 7.36 HBV positive = 27.69 ± 5.67	HBV negative = 37.74 ± 3.22 HBV positive = 37.37 ± 2.91	NR	NR	HBsAg-negative = 1290 HBsAg-positive = 98	HBsAg-negative = 2181 HBsAg-positive = 171
[34]	To (2003) Hong Kong	Retrospective	HBsAg-positive and HBsAg-negative pregnancies	HBsAg-negative = 12,452 HBsAg-positive = 1340	HBsAg-negative = 18 HBsAg-positive = 1	HBsAg-negative = 6 HBsAg-positive = 0	HBsAg-negative = 99 HBsAg-positive = 3	NR	HBsAg-negative = 38.9 ± 2.91 HBsAg-positive = 38.9 ± 2.61	HBsAg-negative = 447 HBsAg-positive = 27	Nulliparity = HBsAg-positive = 622 (46.4) HBsAg-negative = 5952 (47.8) Multiparity = HBsAg-positive = 718 (53.5) HBsAg-negative = 6500 (52.2)	HBsAg-negative = 478 HBsAg-positive = 42	NR
[30]	Mak (2013) Hong Kong	Retrospective cohort	HBsAg-positive and HBsAg-negative pregnancies	HBsAg-negative = 8778 HBsAg-positive = 784	NR	HBsAg-negative = 0 HBsAg-positive = 3	HBsAg-negative = 142 HBsAg-positive = 9	NR	NR	HBsAg-negative = 114 HBsAg-positive = 7	NR	HBsAg-negative = 710 HBsAg-positive = 59	HBsAg-negative = 205 HBsAg-positive = 19
[49]	Huang (2014) China	Cohort	HBsAg-positive and HBsAg-negative pregnancies	HBsAg-negative = 5734 HBsAg-positive = 461	NR	NR	HBsAg-negative = 79 HBsAg-positive = 5	NR	NR	HBsAg-negative = 336 HBsAg-positive = 35	NR	NR	NR
[40]	Zhuang (2017) China	Prospective cohort	HBsAg-positive and HBsAg-negative pregnancies	HBsAg-negative = 35,642 HBsAg-positive = 1113	NR	HBsAg-negative = 1114 HBsAg-positive = 40		NR	NR	NR	NR	HBsAg-negative = 2605 HBsAg-positive = 98	HBsAg-negative = 2767 HBsAg-positive = 108
[48]	Zheng (2021) China	Retrospective cohort	HBsAg-positive and HBsAg-negative pregnancies	HBsAg-negative = 12,813 HBsAg-positive = 1302	NR	NR	HBsAg-negative = 255 HBsAg-positive = 35		39.17 ± 2.06	NR	NR	HBsAg-negative = 1857 HBsAg-positive = 210	HBsAg-negative = 1857 HBsAg-positive = 210
[46]	Zhao (2020) China	Retrospective	HBsAg-positive and HBsAg-negative pregnancies	HBsAg-negative = 29,648 HBsAg-positive = 3789	NR	NR	NR	NR	NR	NR	1 = HBsAg-negative = 11,821 HBsAg-positive = 1394 2 < = HBsAg-negative = 17,805 HBsAg-positive = 2391	HBsAg-negative = 5263 HBsAg-positive = 757	HBsAg-negative = 1336 HBsAg-positive = 190
[39]	Zhao (2022) China		HBsAg-positive and HBsAg-negative pregnancies	HBsAg-negative = 89,686 HBsAg-positive = 10,355	NR	NR	NR	NR	NR	NR	NR	HBsAg-negative = 13,048 HBsAg-positive = 1691	
[35]	Wu (2020) China	Retrospective cohort	HBsAg-positive and HBsAg-negative pregnancies	HBsAg-negative = 18,354 HBsAg-positive = 1146	NR	NR	NR	NR	HBsAg-negative = 38.69 ± 3.56 HBsAg-positive = 37.16 ± 3.92	NR	NR	HBsAg-negative = 3289 HBsAg-positive = 260	HBsAg-negative = 1502 HBsAg-positive = 128
[33]	Sun (2021) China	Cohort	HBsAg-positive and HBsAg-negative pregnancies	HBsAg-negative = 47,855 HBsAg-positive = 1624	HBsAg-negative = 0.5 HBsAg-positive = 0.4	HBsAg-negative = 2.8 HBsAg-positive = 2.7		NR	HBsAg-negative = 38.9 ± 1.9 HBsAg-positive = 38.9 ± 1.6	HBsAg-negative = 1.9 HBsAg-positive = 1.6	NR	HBsAg-negative = 15.5 HBsAg-positive = 13.9	HBsAg-negative = 7.8 HBsAg-positive = 8.1
[24]	Bierhoff (2019) Myanmar-Thailand	Retrospective Cohort	HBsAg-positive and HBsAg-negative pregnancies	HBsAg-negative = 10,338 HBsAg-positive = 687	HBsAg-negative = 44 HBsAg-positive = 3	HBsAg-negative = 1000 HBsAg-positive = 65		NR	HBsAg-negative = 39.0 ± 1.7 HBsAg-positive = 39.1 ± 1.7	HBsAg-negative = 834 HBsAg-positive = 55	NR	HBsAg-negative = 539 HBsAg-positive = 27	NR
[26]	Chen (2022) China	Retrospective Cohort	HBsAg-positive and HBsAg-negative pregnancies	HBsAg-negative = 18,693 HBsAg-positive = 735	HBsAg-negative = 1906 HBsAg-positive = 60	NR	NR	HBsAg-negative = 28.97 (23.43–34.43) HBsAg-positive = 29.72 (23.94–34.91)	NR	HBsAg-negative = 755 HBsAg-positive = 27	NR	HBsAg-negative = 1959 HBsAg-positive = 63	NR
[42]	Cui (2016) China	Prospective cohort	HBsAg-positive and HBsAg-negative pregnancies	HBsAg-negative = 20,491 HBsAg-positive = 513	NR	NR	HBsAg-negative = 216 HBsAg-positive = 4	HBsAg-negative = 27.03 ± 4.19 HBsAg-positive = 27.59 ± 4.02	NR	NR	NR	HBsAg-negative = 232 HBsAg-positive = 6	HBsAg-negative = 1,718 HBsAg-positive = 49
[45]	Wang, Li (2019) China	Retrospective Cohort	HBsAg-positive and HBsAg-negative pregnancies	HBsAg-negative = 7656 HBsAg-positive = 894	NR	NR	NR	NR	NR	HBsAg-negative = 93 HBsAg-positive = 10		HBsAg-negative = 358 HBsAg-positive = 33	HBsAg-negative = 874 HBsAg-positive = 108
[51]	Chen (2015) China	Retrospective Cohort	HBsAg-positive and HBsAg-negative pregnancies	HBsAg-negative = 428 HBsAg-positive = 380	HBsAg-negative = 2 HBsAg-positive = 2	NR	NR	NR	HBsAg-negative = 39.9 ± 1.7 HBsAg-positive = 39.8 ± 1.9	NR	NR	NR	HBsAg-negative = 6 HBsAg-positive = 11
[41]	Cheung (2022) Hong Kong	Retrospective	HBsAg-positive and HBsAg-negative pregnancies	HBsAg-negative = 521 HBsAg-positive = 158	NR	NR	HBsAg-negative = 11 HBsAg-positive = 5	NR	NR	HBsAg-negative = 18 HBsAg-positive = 3	NR	HBsAg-negative = 123 HBsAg-positive = 27	HBsAg-negative = 31 HBsAg-positive = 9
[47]	Peng (2019) china	Retrospective Cohort	HBsAg-positive and HBsAg-negative pregnancies	HBsAg-negative = 964 HBsAg-positive = 964	NR	NR	NR	NR	NR	NR	NR	HBsAg-negative = 16.5% HBsAg-positive = 10.5%	NR
[52]	Liu (2017) china	Retrospective cohort	HBsAg-positive and HBsAg-negative pregnancies	HBsAg-negative = 469,138 HBsAg-positive = 20,827	NR	NR	NR	NR	NR	NR	NR	NR	HBsAg-negative = 24,422 HBsAg-positive = 1344
[44]	Thungsuk (2008) Thailand	Retrospective	HBsAg-positive and HBsAg-negative pregnancies	HBsAg-negative = 170 HBsAg-positive = 154	NR	NR	HBsAg-negative = 0 HBsAg-positive = 2	NR	NR	HBsAg-negative = 3 HBsAg-positive = 3	NR	HBsAg-negative = 4 HBsAg-positive = 5	HBsAg-negative = 10 HBsAg-positive = 19
[43]	Tan (2017) China	Retrospective cohort	HBsAg-positive and HBsAg-negative pregnancies	HBsAg-negative = 21,024 HBsAg-positive = 923	NR	NR	NR	NR	HBsAg-negative = 38.5 HBsAg-positive = 38.3	NR	Nulliparity HBsAg-negative = 16,172 HBsAg-positive = 695 Multiparity HBsAg-negative = 4852 HBsAg-positive = 228	HBsAg-negative = 1696 HBsAg-positive = 109	HBsAg-negative = 1600 HBsAg-positive = 84
[54]	Xu (2021) China	Retrospective cohort	HBsAg-positive and HBsAg-negative pregnancies	HBsAg-negative = 52,094 HBsAg-positive = 2151	NR	NR	NR	NR	NR	NR	0 = HBsAg-negative = 40,464 HBsAg-positive = 1561 1 = HBsAg-negative = 11,337 HBsAg-positive = 565 2 > = HBsAg-negative = 284 HBsAg-positive = 4	NR	HBsAg-negative = 2319 HBsAg-positive = 129
[53]	Salemi (2014)	Retrospective cohort	HBsAg-positive and HBsAg-negative pregnancies	HBsAg-negative = 2,213,722 HBsAg-positive = 3513	NR	NR	NR	NR	NR	NR	Nulliparity HBsAg-negative = 935,227 HBsAg-positive = 1296 Multiparity HBsAg-negative = 1,274,813 HBsAg-positive = 2206	NR	HBsAg-negative = 879,034 HBsAg-positive = 1492

NR Not Reported; GDM: Gastetional Diabetes; R: References, Yrs: Years

### Evaluating the risk of bias

The NOS (Newcastle–Ottawa Quality Assessment Scale) checklist was used to evaluate the quality of the articles. This checklist is designed to assess the quality of cross-sectional studies. Each of these items is given a score of 1 if they are observed in the studies. And the maximum score for each study is 9 points. This step was done independently by two authors (MA and KZ), and in case of disagreement, the cases were referred to the third researcher (YM).

### Statistical analysis

The intended effect size in this meta-analysis was the risk ratio (RR). First, the effect size and the confidence interval were extracted from the studies to perform the analysis. Then, in the desired software for analysis, the logarithm and the standard deviation (SD) of the RR logarithm were calculated, and by combining the logarithm and the standard deviation of the RR logarithm, meta-analysis was conducted. To check the heterogeneity and variance between the selected studies, Cochran’s Q and I2 tests were used. Statistical analysis was performed using STATA 17, and the *P*-value was considered lower than 0.05. Subgroup analyses were performed to determine the main source of heterogeneity in the current meta-analysis based on gestational age, the continent or country of study, and mothers’ age.

## Results

**Fig. 1:**
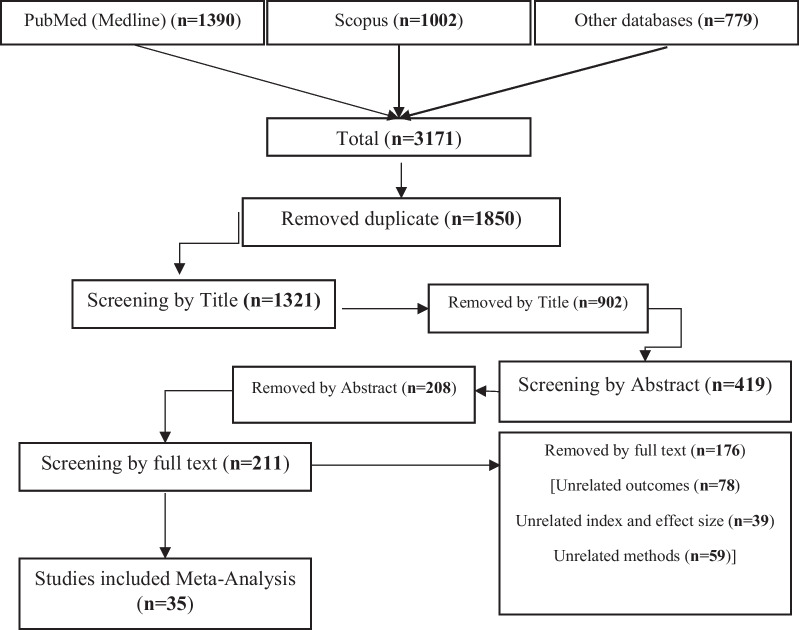
A flow diagram demonstrating the study selection process

In this meta-analysis, after searching and retrieving all articles, 1390 articles in the PubMed database, 1002 articles in the Scopus database, and 779 articles in other relevant databases, including the Cumulated Index to Nursing and Allied Health Literature (CINAHL), SPORT Discuss via the EBSCO interface, Web of Science, and Embase, were retrieved. After removing the duplicates that included 1850 articles, 1321 articles were screened based on the title. In this stage, 902 articles were removed based on the title, and 419 articles entered the screening stage based on the abstract and then the full text. Finally, a total of 384 articles were removed in these steps. Thirty-five cohort studies were selected for meta-analysis and the present study (Table 2) (Fig. 1). The main point was that all selected cohort studies considered chronic HBV and examined its association with the occurrence of pregnancy outcomes.

### Gestational diabetes

**Fig. 2:**
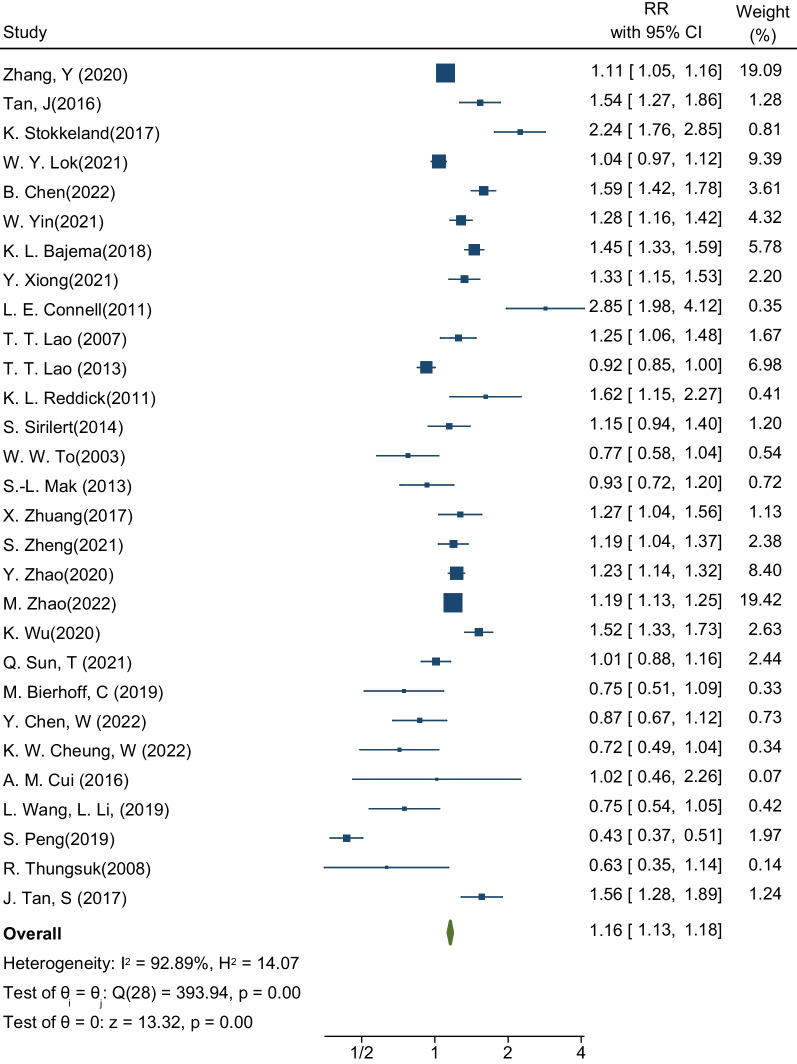
Forest plot of the effect of Hepatitis B Virus on the risk of GDM in pregnant women

The first desired outcome in this meta-analysis was to estimate the incidence of GDM in pregnant women with HBV. The sample size was equal to 32,370,174 pregnant women in a total of 29 studies, of which 121,737 pregnant women were infected with HBV [18, 21–48]. These 29 studies determined the relationship between the presence of HBV infection and the occurrence of GDM. The highest and lowest effect sizes reported in these studies were related to the study by L.E. Connell et al. and the study by S. Peng et al. After pooling the studies, the pooled RR for incident GDM was 1.16. This means that the risk of developing GDM in pregnant women with HBV infection is 1.16 times that of healthy pregnant women (RR: 1.16; 95% CI 1.13–1.18; I square: 92.89%; *P* value: 0.00) (Fig. 2). The analysis of publication bias in this meta-analysis was performed using the Eggers test and reported in Table 3. Based on the results of this test, diffusion bias did not occur in the analysis and combination of studies to investigate the relationship between the presence of HBV infection and the occurrence of GDM (B: -0.89; SE: 0.979; *P*-value: 0.361).

**Table 3:** Meta-analysis of the effect of HBV on the risk of maternal outcomes based on continents, age, and gestational diabetes

Variables	Categories	No. study	Pooled RR (% 95 CI)	Heterogenity Assesment between sudies			Heterogenity Assesment between subgroup		Publication bias assesments		
				I2 (%)	*P* value	Q	Q	Pvalue	B	SE	*P* value
PreEclampsia											
Overall pooled estimate		20	1.10 (1.04–1.16)	92.06	0.00	239.28	–	–	− 0.94	0.661	0.156
Continent	Europe	2	0.71 (0.55–0.92)	0.00	0.89	0.02	53.44	0.00			
	Asia	15	1.24 (1.17–1.32)	92.14	0.00	178.05					
	American	3	0.80 (0.72–0.90)	74.28	0.02	7.78					
Age	< 30	7	1.21 (1.10–1.34)	43.54	0.10	10.63	19.45	0.00			
	> 30	4	0.82 (0.72–0.95)	11.69	0.33	3.40					
Gestational age	< 38	2	0.91 (0.75–1.12)	0.00	0.67	0.18	2.57	0.11			
	> 38	8	1.09 (1.00–1.20)	80.20	0.00	35.35					
GDM											
Overall pooled estimate		29	1.16 (1.13–1.18)	92.89	0.00	393.94	–	–	− 0.89	0.979	0.361
Continent	Europe	1	2.24 (1.76–2.85)	–	–	–	108.08	0.00			
	Asia	24	1.11 (1.09–1.14)	91.58	0.00	273.13					
	American	4	1.54 (1.44–1.65)	76.44	0.01	12.73					
Age	< 30	13	1.12 (1.08–1.16)	94.80	0.00	249.95	1.29	0.26			
	> 30	7	1.16 (1.11–1.21)	86.03	0.00	35.79					
Gestational age	< 38	3	1.38 (1.24–1.54)	65.84	0.05	5.85	14.62	0.00			
	> 38	11	1.11 (1.07–1.14)	85.62	0.00	69.53					
Preterm birth											
Overall pooled estimate		27	1.17 (1.14–1.20)	94.32	0.00	457.57	–	–	− 0.72	0.883	0.417
Continent	Europe	2	1.27 (1.09–1.48)	0.00	0.76	0.09	125.25	0.00			
	Asia	20	1.07 (1.04–1.10)	85.47	0.00	130.79					
	American	5	1.48 (1.41–1.55)	98.01	0.00	201.43					
Age	< 30	12	1.02 (0.97–1.06)	79.03	0.00	52.46	0.02	0.90			
	> 30	6	1.02 (0.96–1.09)	85.73	0.00	35.03					
Gestational age	< 38	3	1.30 (1.17–1.45)	0.00	0.52	1.33	27.62	0.00			
	> 38	8	0.95 (0.91–1.00)	74.05	0.00	26.97					
Eclampsia											
Overall pooled estimate		3	1.48 (0.95–2.29)	0.00	0.87	0.28	–	–	− 0.58	1.749	0.7387
Gestationalhypertension											
Overall pooled estimate		15	0.83 (0.77–0.90)	12.78	0.31	16.05	–	–	0.00	0.604	0.997
Eclampsia + PreEclampsia											
Overall pooled estimate		4	0.85 (0.82–0.89)	48.41	0.12	5.82	–	–	1.60	0.700	0.0220
Abortion											
Overall pooled estimate		2	0.97 (0.71–1.33)	78.96	0.03	4.75	–	–	–	–	–
Neonatal death											
Overall pooled estimate		6	0.83 (0.67–1.03)	0.00	0.95	1.18	–	–	0.22	0.711	0.755

Subgroup analyses by continent, age, and gestational age are reported in Table 3. The results showed that the relationship between the presence of HBV infection and the occurrence of GDM in pregnant women living in Europe is higher than in pregnant women living in Asia and America. But the significant point is that one study is in the European subgroup, which makes the possibility of comparison challenging. If this subgroup analysis is not taken into account and the Asian and American regions are considered, the results show that pregnant women living in the United States (RR: 1.54; 95% CI 1.44–1.65; I square: 76.44%; *P* value: 0.01) have a higher risk of developing HBV infection compared to pregnant women living in Asia (RR: 1.11; 95% CI 1.09–1.14; I square: 91.58%; *P* value: 0.00) (Table 3). Based on maternal age and gestational age by week, the results showed that age over 30 years and gestational age below 38 weeks aggravate the effect of HBV infection on the occurrence of GDM, and the risk of developing GDM is higher in these women (Table 3).

### Preeclampsia

**Fig. 3:**
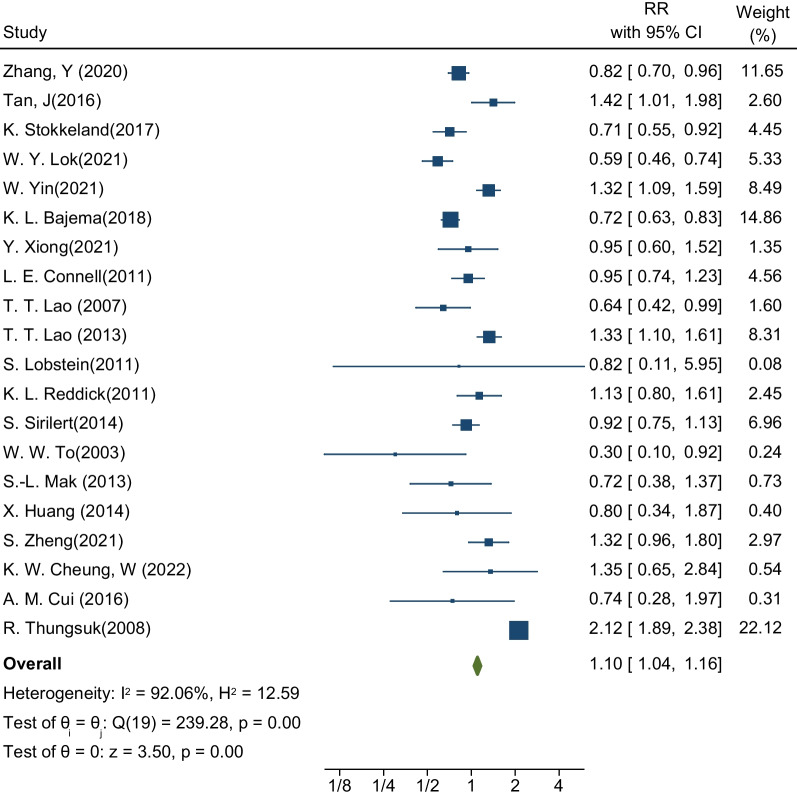
Forest plot of the effect of Hepatitis B Virus on the risk of preeclampsia in pregnant women

The second desired outcome in this meta-analysis was to estimate the risk of preeclampsia in pregnant women with HBV infection. The sample size was equal to 3,217,1538 pregnant women in a total of 20 studies, of which 103,392 were infected with hepatitis [18, 21–23, 27–32, 34, 36–38, 41, 42, 44, 48–50]. These 20 studies determined the relationship between the presence of HBV infection and the occurrence of preeclampsia. The highest and lowest effect sizes reported in these studies, respectively, are related to the study by R. Thungsuk and colleagues (RR: 2.12; % 95 CI 1.89–2.38) and the study by W.W. To et al. (RR: 0.30; % 95 CI 0.10–0.92). After pooling the studies, the pooled RR for preeclampsia was 1.10. This means that the risk of preeclampsia in pregnant women with HBV infection is 1.10 times that of healthy pregnant women (RR: 1.10; % 95 CI 1.04–1.16; I square: 92.06%; *P* value: 0.00) (Fig. 3). The analysis of publication bias in this meta-analysis was performed using the Eggers test and reported in Table 3. Based on the results of this test, diffusion bias did not occur in the analysis and combination of studies to investigate the relationship between the presence of HBV infection and the occurrence of preeclampsia (B: − 0.94; SE: 0.661; *P* value: 0.156).

The results of the subgroup analysis in Table 3 showed a relationship between the presence of HBV infection and the occurrence of preeclampsia in pregnant women living in Asia (RR: 1.24; 95% CI 1.17–1.32; I square: 92.14%; *P* value: 0.00). More than pregnant women living in Europe (RR: 0.71; % 95 CI 0.55–0.92; I square: 0.00%; *P* value: 0.89) and America (RR: 0.80; % 95 CI 0.72–0.90; I square: 74.28%; *P* value: 0.02). The important point is that HBV in pregnant Asian women has a positive and significant association with the occurrence of preeclampsia, while in European and American pregnant women, this relationship is inverse and protective. Based on maternal age and gestational age based on weeks, the results showed that an age below 30 years and a gestational age above 38 weeks aggravate the effect of HBV infection on the occurrence of preeclampsia, and the risk of preeclampsia is higher in these women (Table 3).

### Preterm delivery

**Fig. 4:**
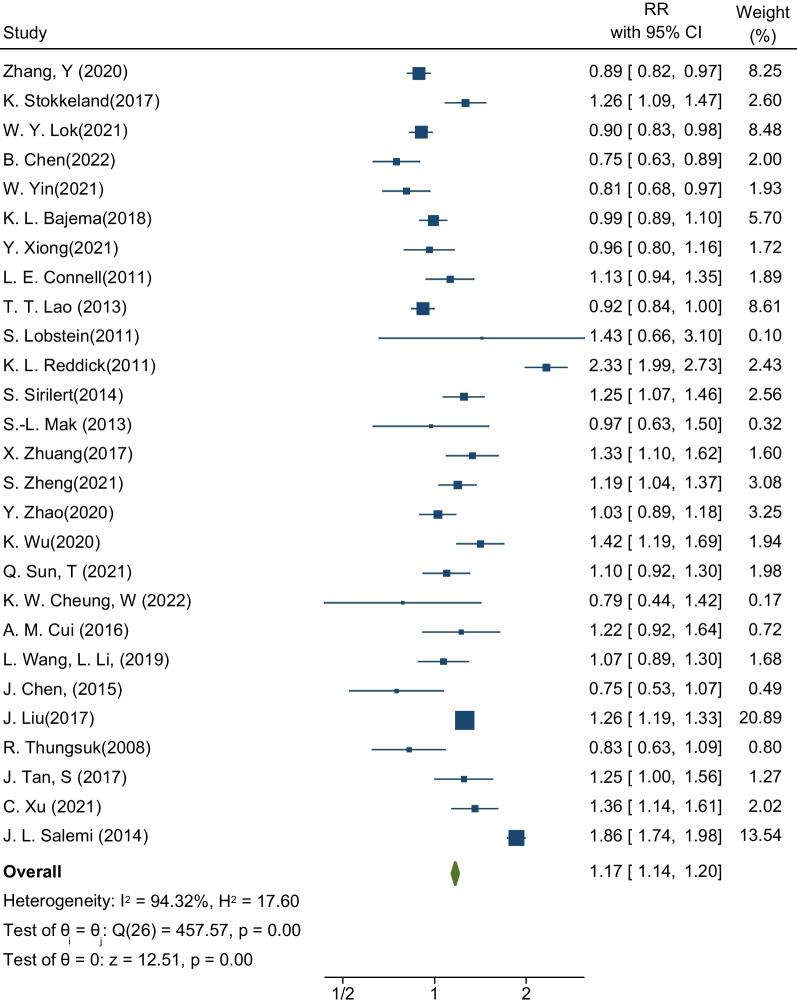
Forest plot of the effect of Hepatitis B Virus on the risk of preterm birth in pregnant women

The third desired outcome in this meta-analysis was the estimation of the risk of premature delivery in pregnant women with HBV infection. The sample size was equal to 34,950,154 pregnant women in a total of 27 studies, of which 132,441 pregnant women were infected with hepatitis [21, 23, 25, 27–33, 35–38, 40–46, 48, 50–54]. These 27 studies determined the relationship between the presence of HBV infection and the occurrence of premature birth. The highest and lowest effect sizes reported in these studies are respectively related to the study of L. Reddick K. et al. (RR: 2.33; 95% CI 1.99–2.73) and the study of B. Chen et al. (RR: 0.75; % 95 CI 0.63–0.89). After pooling the studies, the pooled RR for preterm delivery was 1.17. This means that the risk of premature delivery in pregnant women with HBV infection is 1.17 times that of healthy pregnant women (RR: 1.17; % 95 CI 1.14–1.20; I square: 94.32%; *P* value: 0.00) (Fig. 4). The analysis of publication bias in this meta-analysis was performed using the Eggers test and reported in Table 3. Based on the results of this test, diffusion bias did not occur in the analysis and combination of studies to investigate the relationship between the presence of HBV infection and the occurrence of premature birth (B: − 0.72; SE: 0.883; *P* value: 0.417).

The results of the subgroup analysis in Table 3 showed that the relationship between the presence of HBV infection and the occurrence of premature birth in pregnant women living in the United States (RR: 1.48; 95% CI 1.41–1.55; I square: 98.01%; *P* value: 0.00) is greater than that in pregnant women living in Europe (RR: 1.27; % 95 CI 1.09–1.48; I square: 94.12%; *P* value: 0.00) and Asia (RR: 1.07; % 95 CI 1.04–1.10; I square: 85.47%; *P* value: 0.00). Based on maternal age and gestational age by week, the results showed that gestational age lower than 38 weeks aggravates the effect of HBV infection on the occurrence of premature birth, and the risk of premature birth is higher in these women (Table 3).

### Other outcomes

Other outcomes examined in this meta-analysis included eclampsia, gestational hypertension, miscarriage, and neonatal death. The meta-analysis results showed that the risk of eclampsia in pregnant women with HBV infection was equal to 1.48 (RR: 1.48; 95% CI 0.95–2.29; I square: 0.00%; *P* value: 0.87), but it was not statistically significant. If the risk of gestational hypertension (RR: 0.83; % 95 CI 0.77–0.90; I square: 12.78%; *P* value: 0.31), miscarriage (RR: 0.97; % 95 CI 0.71–1.33; I square): 78.96%; *P* value: 0.03), and neonatal death (RR: 0.83; % 95 CI 0.67–1.03; I square: 0.00%; *P* value: 0.95) in pregnant women with HBV infection was less than one. These results are reported in the Additional file 1.

## Discussion

The purpose of the present meta-analysis was to determine the relationship between HBV infection during pregnancy and the occurrence of pregnancy outcomes such as preeclampsia, premature birth, gestational diabetes, abortion, eclampsia, and hypertension during pregnancy. The results showed that the presence of HBV infection could increase the risk of adverse pregnancy outcomes such as preeclampsia, premature birth, gestational diabetes, abortion, eclampsia, and hypertension.

Preeclampsia can be considered one of the most common pregnancy complications in the last months of pregnancy (usually from the 20th week of pregnancy to 7 days after delivery), which is observed in 5% of pregnant women. Preeclampsia has no specific symptoms and is dangerous for the fetus and the mother. The contraction of blood vessels causes this disease and, as a result, leads to an increase in blood pressure and a decrease in blood flow in fetal organs such as the liver, kidney, and brain. This reduction in blood flow in the uterus leads to problems for the fetus, such as reduced growth, reduced amniotic fluid, etc. The current meta-analysis showed that the presence of HBV infection could increase the risk of preeclampsia in pregnant women by 10% compared to pregnant women without HBV infection. Meta-analysis studies are conducted to determine the more accurate and error-free effect and relationship between two important factors. These studies can control many possible errors in the relationship between exposure and the desired outcome. The important point in studying the relationship between the presence of HBV infection and the occurrence of preeclampsia in pregnant women was the difference in the pathophysiology and epidemiology of preeclampsia and HBV infection in early studies conducted in the world. In addition, the statistical population and method of care for each of these conditions differed in these studies. Other infections, such as parasitic, viral, and bacterial infections, may have a significant impact on the incidence and prevalence of pre-eclampsia in pregnant women, and the presence of other blood-borne and sexually transmitted infections, particularly hepatitis C and HIV/AIDS, may also increase susceptibility to HBV infection. As a result, it is likely that the presence of these conditions and other infections predisposes women to pre-eclampsia, with HBV acting only as an aggravating or enabling factor [23, 25, 28, 55, 56]. This factor was one of the main reasons for the high heterogeneity in the analysis of the relationship between the presence of HBV infection and the occurrence of preeclampsia.

Subgroup analyses in determining the relationship between HBV infection and the occurrence of preeclampsia in pregnant women showed that the risk of infection is higher in Asian women. This result confirmed the difference in the effect of HBV on the occurrence of preeclampsia in different geographical regions, because in other regions such as Europe or America, the relationship between HBV infection and the occurrence of preeclampsia was an inverse or protective relationship. The main reason for this is the difference in culture, the way services related to prenatal care before, during, and after pregnancy are provided and received, and, most importantly, the prevalence of other infections that are effective in Asian countries or other locations. Finally, there are plausible reasons in clinical texts for the association between HBV and the development of pre-eclampsia. In general, HBV infection has been associated with an increased risk of atherosclerosis in pregnant women [56–60]. Furthermore, preeclampsia (marked by maternal endothelial dysfunction) may result in an imbalance of angiogenic, anti-angiogenic, and proangiogenic substances such as vascular endothelial growth factor [55, 61–65]. According to previous research, there is a considerable link between HBV infection and insulin resistance, thrombocytopenia, obesity, and kidney damage or proteinuria [66–68]. The interplay of these illnesses and HBV could explain the link between HBV and preeclampsia.

In the present meta-analysis, results showed that the presence of HBV infection increases the risk of gestational diabetes in pregnant women by 16% compared to women without HBV infection. Also, the risk of developing gestational diabetes in Asian women with HBV infection was lower than that of American women. This difference can be attributed to the importance of the issue of pregnancy and the difference in receiving services related to gestational diabetes screening in the Asian region and Asian countries. In addition, the different diet (especially in Southeast Asian regions) and the way of doing physical activity can be considered other reasons for this difference [69, 70]. Another point that can justify this relationship between Asian pregnant women is the difference in the prevalence of HBV infection in different regions, especially in different Asian and American countries [71, 72]. Of course, the various studies conducted in this field and the results of this meta-analysis confirm the fact that to more accurately determine the relationship between HBV infection and the occurrence of gestational diabetes, there is a need to conduct more studies taking into account the prevalence of HBV, the presence of chronic disease, and the background another is genetic factors and environmental factors such as nutritional and non-nutritive behaviors (smoking, alcohol, unprotected sex, etc.) [37, 73, 74]. An important factor that needs to be investigated in this connection is the body mass index of pregnant women, which can disrupt the relationship between HBV infection and the occurrence of gestational diabetes as an important confounding factor. In addition, the presence of other factors, such as high blood pressure, can also be one of the other factors influencing the relationship between HBV infection and the occurrence of gestational diabetes. These causes can be related to the occurrence of pre-eclampsia, metabolic syndrome, and then GDM [18, 28, 75, 76]. The current meta-analysis used a search technique that lasted through February 2023, and all retrieved studies were examined and screened. The inclusion of a defined selection of cohort studies, as well as the consideration of a specific time span for analysis and reporting, distinguishes this meta-analysis from review studies. Because the goal of this meta-analysis was to look at the relationship between hepatitis B infection and pregnancy outcomes, cohort studies were deemed the best primary research design for determining the relationship without taking into account interventions. This was one of the most significant distinctions between this meta-analysis and earlier research. In contrast, previous meta-analyses did not include all pregnancy outcomes. The outcomes of gestational diabetes and preterm birth were not included in the study by Karamati et al. [77], and the outcomes of gestational diabetes, pre-eclampsia, preterm birth, and miscarriage were not included in the analysis and review by Oliviera et al. [78] These outcomes were evaluated and analyzed in the current meta-analysis.

The important point of this meta-analysis was to estimate the effect size with high accuracy. Although the degree of heterogeneity in the estimated effect size was high, this degree of heterogeneity was indicative of statistical heterogeneity as determined by the I square index and Cochrane’s Q test. The important point was the absence of clinical heterogeneity or its presence at an acceptable level, which is interpreted by the estimated confidence intervals. All the confidence intervals obtained for the desired relationships in the present meta-analysis were narrow, which indicated high precision in the analysis. On the other hand, the narrow confidence interval is one of most important items in clinical interpretation and clinical justification of communication. For example, to determine the relationship between HBV infection and gestational diabetes, the estimated effect size was 1.16 with a confidence interval of 1.13 to 1.18. Despite the heterogeneity rate of 92.7%, the calculated confidence interval is very narrow, which indicates the existence of a sufficient sample and number of studies to determine the relationship and confirms the accuracy of the calculated relationship. Another strength of the present meta-analysis was the subgroup analysis based on the variables of geographic regions, gestational age, and the age of the pregnant mother, which to some extent, shows the role of confounding and other influencing variables in the relationship between HBV infection and pregnancy outcomes. The results of this meta-analysis can be very effective in developing or updating clinical guidelines.

One of the weaknesses or limitations of the present meta-analysis is the failure to perform subgroup analyses based on important variables such as body mass index, the presence of other infections (HIV/AIDS, hepatitis C infection), other underlying diseases (hypertension, genetics) pointed out that due to the lack of reporting of primary studies in their results, they were not included in the results of the present meta-analysis. We suggest that future studies be conducted to determine the relationship between HBV infection and the occurrence of pregnancy outcomes by considering these variables.

## Conclusion

This meta-analysis found that hepatitis B infection during pregnancy may be associated with an increased risk of gestational diabetes, preterm delivery, pre-eclampsia, and eclampsia. However, confirmation of this association, as well as the specific biological pathways involved in the association between HBV infection and pregnancy outcomes, requires further investigation. As a result, it is crucial to enhance programs and healthcare services for women in society, focusing on the promotion of screening, care, and treatment programs for infectious diseases, particularly HBV. These efforts should be implemented across various communities, with particular emphasis on developing societies.

### Supplementary Information

**Additional file 1.** Supplementary Figures.

## Data Availability

Data and materials are available within the complementary materials, and further information can be available by request to the corresponding author.

## Abbreviations

CI: Confidence interval
I^2^: I square
RR: Risk ratio
PRISMA: Preferred reporting items for systematic reviews and meta-analyses
GDM: Gastational diabetes
CINAHL: Cumulated index to nursing and allied health literature
HBeAg: Hepatitis B e antigen
CccDNA: Covalently closed circular DNA
HBV: Hepatitis B virus
