# Supplementary material for: The effect of hepatitis B virus on the risk of pregnancy outcomes: a systematic review and meta-analysis of cohort studies
Source: Virol J. 2023 Sep 14;20:213. doi: 10.1186/s12985-023-02182-0 (PMC10500763; doi:10.1186/s12985-023-02182-0)
Supplement: Supplementary file 1 — Additional file 1. Supplementary Figures. [file 12985_2023_2182_MOESM1_ESM.docx]

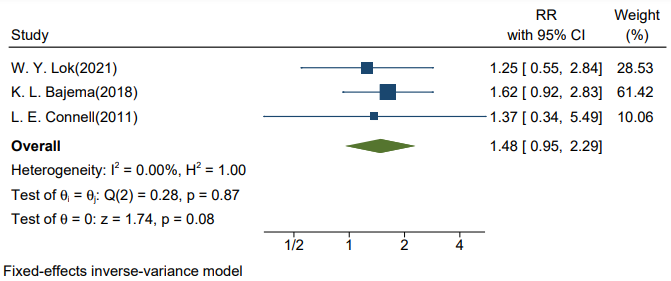


Figure 1: Forest plot of the effect of Hepatitis B Virus on the risk of eclampsia in pregnant women


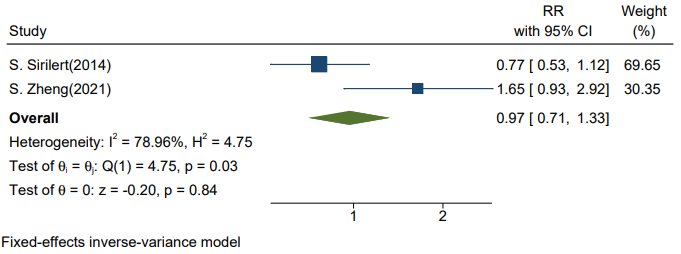


Figure 2: Forest plot of the effect of Hepatitis B Virus on the risk of abortion in pregnant women


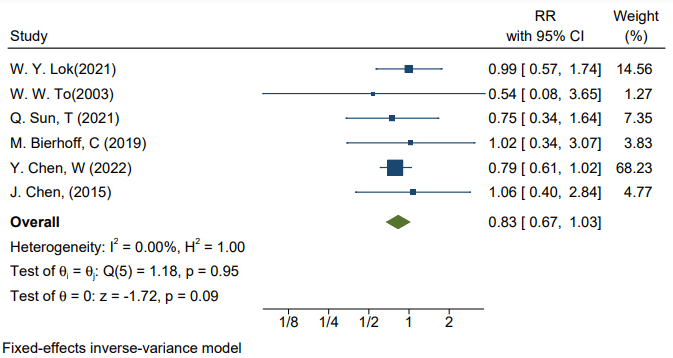


Figure 3: Forest plot of the effect of Hepatitis B Virus on the risk of neonatal death in pregnant women

Figure 4: Forest plot of the effect of Hepatitis B Virus on the risk of gestational hypertension in pregnant women
